# Adult-onset foveomacular vitelliform dystrophy: epidemiology, pathophysiology, imaging, and prognosis

**DOI:** 10.3389/fopht.2023.1237788

**Published:** 2023-08-10

**Authors:** Grace E. Nipp, Terry Lee, Kubra Sarici, Goldis Malek, Majda Hadziahmetovic

**Affiliations:** ^1^ School of Medicine, Duke University, Durham, NC, United States; ^2^ Department of Ophthalmology, Duke University Medical Center, Durham, NC, United States; ^3^ Department of Pathology, Duke University Medical Center, Durham, NC, United States

**Keywords:** adult-onset foveomacular dystrophy, AOFVD, vitelliform dystrophy, acquired vitelliform lesions, AVL

## Abstract

Adult-onset foveomacular dystrophy (AOFVD) is a retinal pattern dystrophy that may affect up to 1 in 7,400 individuals. There is much that is unknown regarding this disease’s epidemiology, risk factors for development, and rate of progression through its four stages. Advancements in retinal imaging over the past 15 years have enabled improved characterization of the different stages of AOFVD. These imaging advancements also offer new ways of differentiating AOFVD from phenotypically similar retinal diseases like age-related macular degeneration and Best disease. This review synthesizes the most recent discoveries regarding imaging correlates within AOFVD as well as risk factors for the development of AOFVD, complications of AOFVD, and treatment options. Our aim is to provide ophthalmologists a succinct resource so that they may offer clarity, guidance, and appropriate monitoring and treatments for their patients with suspected AOFVD.

## 1 Introduction

“A peculiar central macular dystrophy” was first described in 1974 by J. Donald Gass in a case series of nine patients who all held disparate symptoms but demonstrated a common finding on fundoscopy: a symmetrical, elevated, yellow subretinal lesion with a central pigmented spot in the fovea (1). This “peculiar” dystrophy, now known as adult-onset foveomacular vitelliform dystrophy (AOFVD), has since been studied by many groups worldwide, but there remains much about this retinal disease that is incompletely understood.

Even a consensus on the disease’s name took several decades. Since its first description by Gass, it has been referred to as adult vitelliform macular degeneration (2–4), adult macular vitelliform degeneration (5), pseudovitelliform macular degeneration (6), adult-onset foveomacular pigment epithelial dystrophy (7, 8) adult foveomacular vitelliform dystrophy (9, 10), and adult vitelliform macular dystrophy (11–13). The name adult-onset foveomacular vitelliform dystrophy was first used by Battaglia Parodi et al. in 1996 (14) but did not gain widespread acceptance until the 21^st^ century. Further, within clinical practice, many physicians still use older terms such as adult vitelliform macular dystrophy and adult-onset foveomacular pigment epithelial dystrophy.

AOFVD is included in a broader group of retinal pathologies known as pattern dystrophies, in which pigment accumulates in the retinal pigment epithelium (RPE) in the macula, most often bilaterally. Other pattern dystrophies include butterfly-shaped pigment dystrophy, reticular dystrophy, multifocal pattern dystrophy, and fundus pulverulentus. There is debate as to whether AOFVD strictly belongs in this group of pattern dystrophies, as it does not have the clear autosomal dominant (AD) inheritance seen in other dystrophies, nor are all eyes with AOFVD associated strictly with pigment deposition and disruption of the RPE (3, 5).

AOFVD is characterized by the presence of subfoveal vitelliform material detected by fundus examination as well as multimodal imaging. The morphology of the vitelliform lesion changes as the disease progresses, which informs the staging of the disease (3, 5, 13, 15). However, AOFVD generally progresses slowly, with most patients experiencing a relatively slight decrease in best-corrected visual acuity (BCVA) (3, 5). Significant reduction in BCVA is associated with the progression to macular atrophy or development of choroidal neovascularization (CNV) (3, 5). Newer imaging modalities that have become available over the past 15 years, such as spectral domain optical coherence tomography (SD-OCT) and OCT angiography (OCTA), continue to expand our ability to diagnose and characterize AOFVD and assess for these sequelae.

Despite progress in imaging modalities, AOFVD remains a misunderstood disease frequently misdiagnosed as age-related macular degeneration (AMD), resulting in unnecessary treatment. Fortunately, the advent of novel imaging techniques over the past decade has allowed for greater multimodal imaging characterization of this disease that has significantly improved the understanding and diagnosis of AOFVD. This paper aims to summarize these most recent advancements in AOFVD and to provide an updated and concise review of the diagnostic standards and relevant imaging findings associated with AOFVD, its complications, pathophysiology, and current treatment standards.

## 2 Epidemiology and pathophysiology

### 2.1 Epidemiology

Though Gass postulated that the average age of onset might be between the 3^rd^ and 5^th^ decades of life, subsequent studies have suggested a much later onset, with most patients not developing symptoms or being diagnosed until they are between 50 to 70 years of age (1, 3, 5, 13, 16). However, the age of onset is highly variable, with studies frequently reporting ranges of 30 years of age up to 80 years (3, 5). Rates of diagnosis between men and women are not regularly reported, but three studies reported a slightly greater proportion of their cohorts being female, between 57% and 66% (5, 16, 17). Other studies report an equal distribution between male and female participants (3).

Race and ethnicity trends have also not been well studied, with most studies failing to describe the race or ethnicity of their participants. However, studies on AOFVD are published from diverse geographic locations worldwide, including China, Japan, the U.S., South America, the Middle East, and across Europe (18–22). This suggests that the disease can be found in most populations.

AOFVD is known to be a rare disease, and its prevalence has seldom been reported. Dalvin et al. reported the prevalence of AOFVD in Olmsted County, Minnesota as being between 1 in 7400 to 8200 individuals (23). Prevalence otherwise remains undescribed. Misdiagnosis of AOFVD or miscoding of the disease as AMD in the electronic health record can make it difficult to assess its true prevalence.

### 2.2 Genetic mutations and inheritance

Early articles exploring AOFVD suggested an AD inheritance pattern (1, 7, 12). However, it has since become clear that most cases of AOFVD are sporadic and do not follow a clear inheritance pattern (3, 5, 6). That said, several genes have been associated with AOFVD, including *PRPH2*, *BEST1*, *IMPG1*, and *IMPG2*.

#### 2.2.1 PRPH2

The *PRPH2* gene was identified in 1998 (24, 25). It encodes for peripherin 2, a glycoprotein located on the surface of photoreceptors in the retina. Peripherin 2 is thought to play a critical role in the formation and stabilization of rods and cone outer segment discs, functioning as an adhesion molecule (26). Though mutations in *PRPH2* are the most common gene mutations identified in AOFVD patients, they account for only 2-18% of all patients with AOFVD (26, 27). The exon region of *PRPH2* is polymorphic, and it has been hypothesized that individuals carrying single nucleotide polymorphism (SNP) variants of *PRPH2* are at increased risk of developing AOFVD (28). However, a study by Grunin et al. found no significant association between 14 SNPs and AOFVD in their study population (29). Cavdarli et al. concluded that mutations in *PRPH2* are not present in many patients, though mutations in this gene may act as a predisposing factor for the development of AOFVD (30).

#### 2.2.2 BEST1

*VMD2* mutations were first associated with AOFVD in 1998. Since then, *VMD2* has been renamed *BEST1* (25). *BEST1* mutations have been associated with Best vitelliform macular dystrophy, AOFVD, AD vitreoretinochoroidopathy, autosomal recessive bestrophinopathy, and retinitis pigmentosa (31). The gene encodes for bestrophin-1, a transmembrane protein primarily expressed in the RPE. It acts as an ion channel and plays a role in intracellular calcium signaling (32, 33). It is thought that compromise of the RPE-photoreceptor interface occurs secondary to a mutation in *BEST1*, which may increase the vulnerability of the rods and cones to biochemical changes. This compromise eventually results in areas of subretinal fluid accumulation and the loss of RPE and overlying photoreceptors (34). Studies have demonstrated that different mutations within *BEST1* impart varying levels of disruption in calcium channel function, which may correspond with the severity of the disease (26, 31, 35). Because of this, some hypothesize that AOFVD patients with certain *BEST1* mutations may actually possess a mild version of Best disease with later onset (26, 31, 35).

#### 2.2.3 IMPG1 and IMPG2

The *Interphotoreceptor Matrix Proteoglycan 1* (*IMPG1*) and *2* (*IMPG2*) genes were more recently identified as genes associated with AOFVD, with *IMPG1* being identified in 2013 (36) and *IMPG2* being identified the following year (37). These two genes encode for proteins that are secreted into the extracellular matrix in the retina and play a role in retinal adhesion (38). Meunier et al. reported the frequency of IMPG1 and IMPG2 mutations among familial AOFVD patients who do not have *PRPH2* or *BEST1* mutations as being 4 in 49, or approximately 8%. Thus, its prevalence among all AOFVD patients is likely less than 8% (37). A recent case report supports that *IMPG2* mutations can be detected in patients with AOFVD with no family history of the disease (39).

### 2.3 Histopathology

Histopathologic examination of AOFVD remains sparse despite the important role it plays in revealing disease etiology and progression. No new histologic papers specific to AOFVD have been published since 2003. In total there are five papers that discuss AOFVD histopathology: Gass’s original paper, Patrinely et al., Jaffe and Schatz, Dubovy et al., and Arnold et al. (1, 8, 40–42)

Disruption of photoreceptors in the foveal region is common across all studies. Some show RPE hypertrophy in the macula, while others demonstrate peripheral hypertrophy (8, 40). Across all these papers, there is a general consensus that AOFVD is a clinical spectrum of disease that results from the disordered metabolism of RPE cells, resulting in the accumulation of material in the subretinal space. As the material accumulates, RPE atrophy may ensue, triggering changes in the overlying retina.

Both Gass and Patrinely et al. noted disruption and atrophy of photoreceptors in the foveal region. Gass demonstrated hypertrophy of the RPE in the macula (corresponding to hyperpigmentation on fundoscopy), while Patrinely et al. saw peripheral hypertrophy of RPE with atrophy of the RPE in the macula. Both studies also demonstrated pigment-laden macrophages that had migrated from the RPE into the overlying retina and periodic-acid Schiff staining material that had accumulated between the RPE and Bruch’s membrane. Both reported the presence of calcific bodies, with Gass reporting the bodies as being between the RPE and Bruch’s membrane and Patrinely et al. reporting them as extending into the retina.

Where Gass and Patrinely et al. diverge is in the reporting of lipofuscin. Lipofuscin is a pigment produced through oxidative damage and as a metabolic product. It is seen in other retinal diseases such as Best disease, fundus flavimaculatus, Stargardt disease, and some melanomas (40). Lipofuscin could contribute to the yellow appearance of the vitelliform lesion (40). Patrinely et al. noted a large amount of lipofuscin within abnormal RPE cells and macrophages whereas Gass reported minimal lipofuscin. Similarly, Jaffe, Schatz and Arnold et al. did not find evidence of excessive lipofuscin. However, Dubovy et al. did find a significant amount of lipofuscin in the RPE and macrophages in 3 of their cases. Some hypothesize that the amount of lipofuscin in the histopathologic samples correlates with the stage of disease, with earlier vitelliform lesions possessing greater amounts and later stage disease demonstrating reabsorption of the lipofuscin (42).

## 3 Clinical diagnosis, staging, and imaging correlates

**Figure 1:**
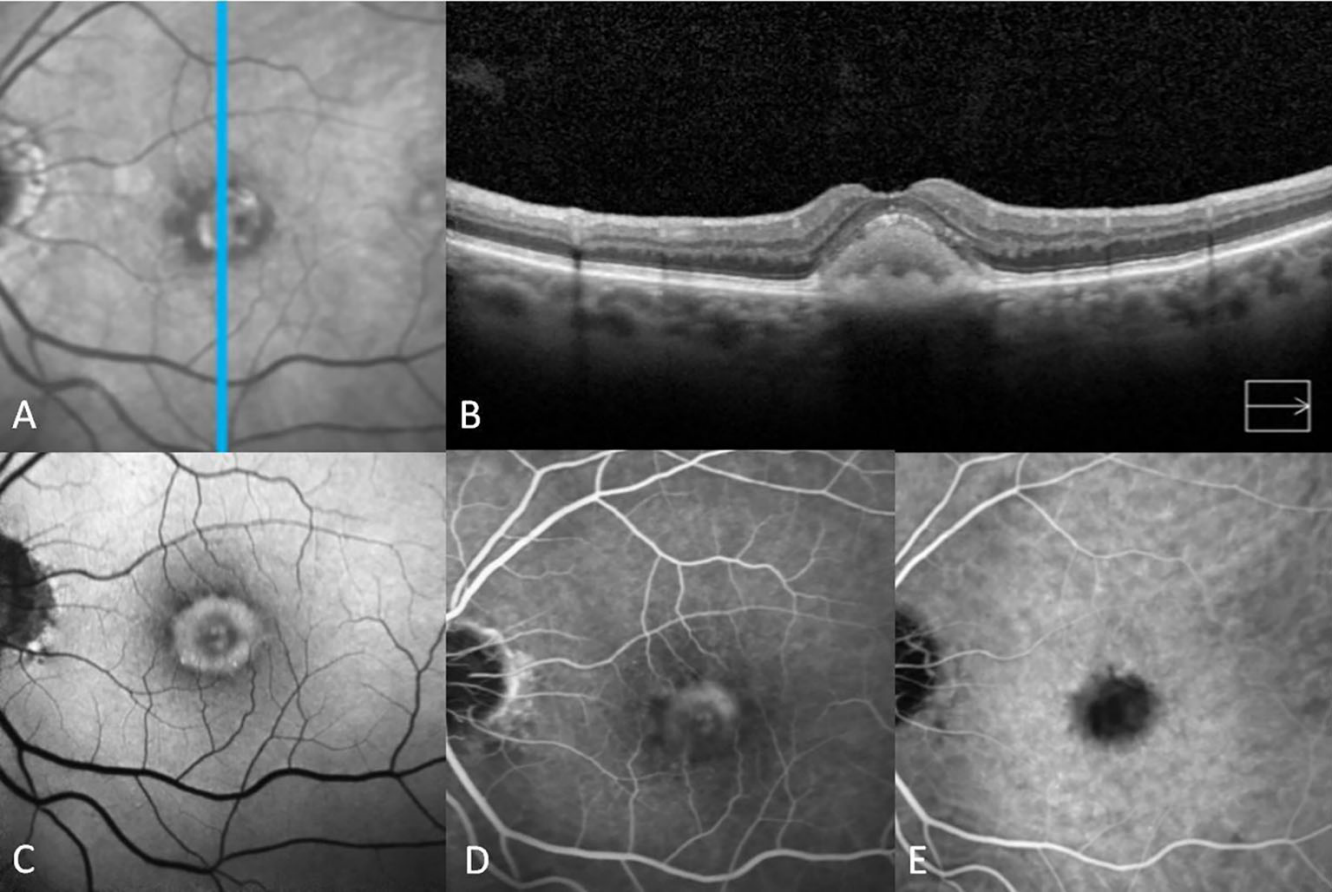
Multimodal imaging features of adult-onset foveomacular vitelliform dystrophy in the vitelliform stage. The infrared image shows a central foveal white area surrounded by a mottled black ring **(A)**. Vertical spectral-domain optical coherence tomography shows relatively diffuse homogeneous subretinal hyperreflective material (SHRM) with a smooth surface **(B)**. Fundus autofluorescence imaging shows central hyperautofluorescence surrounded by a ring of hypo-autofluorescence **(C)**. Fluorescein angiography reveals staining without leakage **(D)**. Indocyanine green angiography shows hypocyanescence at the location of the vitelliform material in accordance with blockage in all phases **(E)**.

**Figure 2:**
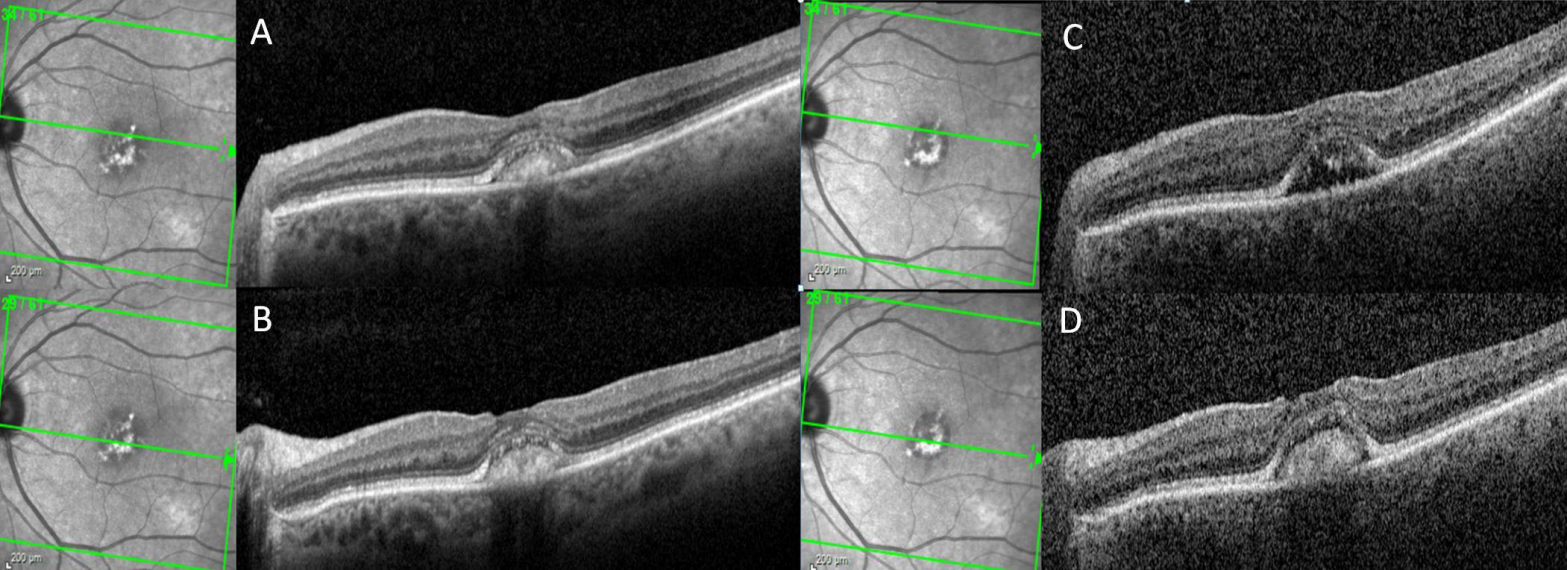
A patient with adult-onset foveomacular vitelliform dystrophy progresses from the vitelliform stage to pseudohypopyon stage over the course of 1 year. On horizontal spectral-domain optical coherence tomography (SD-OCT), vitelliform material is observed across the fovea both at baseline and at the 12-month follow-up visit **(A, B)**. Horizontal SD-OCT from the superior aspect of the vitelliform lesion demonstrates resolution of the vitelliform material and left a hyporeflective cavitation at the 12-month follow-up visit compared to the baseline visit **(C, D)**.

**Figure 3:**
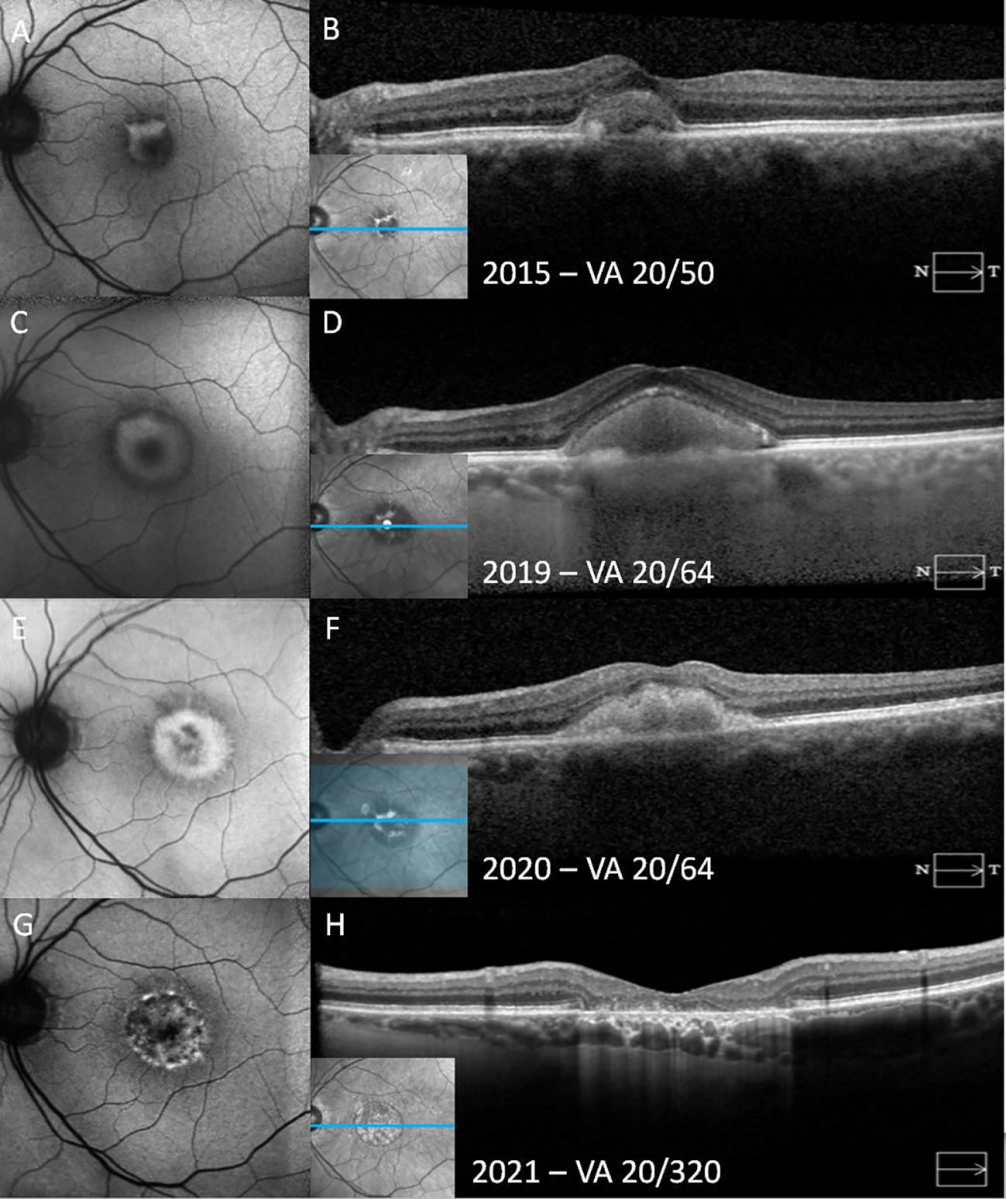
A patient with adult-onset foveomacular vitelliform dystrophy progresses from the vitelliform stage to macular atrophy over the course of 6 years. In the vitelliform stage, the fundus autofluorescence (FAF) shows a C-shaped hyperautofluorescent area **(A)**. Horizontal spectral-domain optical coherence tomography (SD-OCT) demonstrates subretinal hyperreflective materials (SHRM) overlying elongated outersegments and a thick granular retinal pigment epithelium (RPE) at its base **(B)**. Over time, the vitelliform material concentrically increases in size on FAF, accompanied by increased SHRM on SD-OCT **(C, D)**. FAF demonstrates progression to the vitelliruptive stage, with the hyperautofluorescent area becoming heterogeneous and hypoautofluorescent in the center of the lesion **(E)**. In SD-OCT, the surface of SHRM has become lobular, and its homogeneity has deteriorated **(F)**. In the atrophic stage, on FAF, previously hyperautofluorescent areas have become mottled with heterogeneous hypoautofluorescence, corresponding to RPE atrophy. The atrophic area is surrounded by a hyperautofluorescent ring **(G)**. SD-OCT shows atrophy of all outer retinal layers, including the outer nuclear layer, photoreceptor layer, and the RPE with hypertransmission through the corresponding areas **(H)**.

AOFVD progresses through four clinical stages, first described by Querques et al. in 2011 using SD-OCT (16). The clinical stages are defined as:

a. Stage I, vitelliform stage: This stage is defined by the classic vitelliform “egg-yolk” lesion, which is visible on fundoscopy (**Figure 1**).
b. Stage II, pseudohypopyon stage: This stage is characterized by the layering of lipofuscin within the vitelliform lesion (**Figure 2**).
c. Stage III, vitelliruptive stage: During this stage, the vitelliform lesion is broken up and reabsorbed. The prior acquired vitelliform lesion may take on the “scrambled egg” on fundoscopy (**Figure 3**).
d. Stage IV, atrophic stage: This final stage presents following the resorption of vitelliform material. However, not all patients with resorption of the acquired vitelliform lesion (AVL) will progress towards atrophy (**Figure 3**).

Below, we describe the major findings seen with each imaging modality, as well as the major findings associated with each stage of AOFVD, where such information is available. **Table 1** provides a succinct summary of the findings associated with each stage across imaging modalities.

**Table 1:** The four stages of adult-onset foveomacular vitelliform dystrophy and their respective multimodal imaging correlates.

	1. Vitelliform	2. Pseudohypopyon		3. Vitelliruptive	4. Atrophic
Color Fundus Photography	“Egg-yolk” appearance Lesion is yellowish-white, rounded, regular in shape, centered on fovea (3)	Unchanged as compared to vitelliform stage (43)		“Scrambled egg” appearance Yellowish lesion appears to break apart, is less regular in shape (43)	Visible choroidal blood vessels Pale fundus (44)
Fundus Autofluorescence	Entire lesion is hyperautofluorescent (13)	Hyperautofluorescent inferior half, hypoautofluorescent superior half (45)		Hypoauto-fluorescent (45, 46)	Hypoauto-fluorscent (45, 46)
Fluorescein Angiography	Non-fluorescent central spot in lesion Hyperfluorescent spoked ring around lesion (3, 10, 13, 47)	Less hyperfluorescent “Stars-in-the-sky” appearance (48)		Persistent hyper-fluorescence “Stars-in-the-sky” appearance (48)	Late-stage hyperfluorescence of atrophic area (44)
Spectral Domain Optical Coherence Tomography	Intralesional cuticular Drusen at RPE/Bruch’s membrane complex is common Subretinal drusenoid deposits Disruption of the ellipsoid zone				
	Dome-shaped homogeneous SHRM (10, 16, 43)	Two zones of the vitelliform lesion (43). * Upper zone is hyporeflective with a few clumps of SHRM * Lower zone features homogenous SHRM Normal overlying retina * Intraretinal pseudocysts may be present		Fragmented vitelliform lesion with mix of hyper- and hyporeflective spaces with overlying photoreceptor loss (16) As lesion progresses, SHRM may “clump” and resolve along RPE layer (43).	Widespread loss of photoreceptor layers and RPE atrophy. Corresponds with cRORA definition (44)
Optical Coherence Tomography Angiography	Increased subfoveal choroidal thickness, particularly as compared to AMD patients				
	Reduced blood flow in superficial and deep choroid plexus in areas corresponding to lesion (19, 49, 50) Reduction in apparent choriocapillaris vessel density (19, 49, 51, 52)	Reduced blood flow in superficial and deep choroid plexus in areas corresponding to lesion (19, 49, 50) Reduction in apparent choriocapillaris vessel density (19, 49, 51, 52)		Increased choriocapillaris vessel density (19)	Increased choriocapillaris vessel density (19)

SHRM, subretinal hyperreflective material; cRORA, complete retinal pigmental epithelium and outer retinal atrophy; AMD, age-related macular degeneration.

### 3.1 Color fundus photography

As mentioned, the classic AVL associated with AOFVD fits an “egg-yolk” appearance: a yellow, elevated lesion within the fovea with a central pigmented spot. At times, the central spot may not appear pigmented but, instead, appears as a separate elevated “figure” underlying the lesion (3). The vitelliform lesion may also be described as a pigmented clump with hypopigmented halos (3), though some groups choose to exclude these morphologies from AOFVD clinical studies. The AVL associated with AOFVD is rounded and regular in shape, mostly centered on the fovea and sometimes at the perifoveal area. Drusen can be observed surrounding the AVL, though they are less prominent than in AMD (45). The AVLs associated with AOFVD are generally less than 1 disc diameter in size (13).

Studies have demonstrated that most patients diagnosed with AOFVD (either with the yellow lesion type or pigmented type) will possess lesions bilaterally (3, 13, 45, 46). However, bilateral lesions are not required for diagnosis. Additionally, despite the presence of bilateral lesions, the progression of the lesions may not be symmetric; the two eyes may be observed at different stages (3).

The vitelliform and pseudohypopyon stages are indistinguishable on color fundus photography. During the vitelliruptive stage, the AVL may take on a “scrambled egg” appearance due to the patchy reabsorption of the lesion. This correlates on fundoscopy to a less homogeneous lesion with the dispersion of the raised, yellow material without corresponding atrophy or subretinal fibrosis (43).

### 3.2 Fundus autofluorescence

The vitelliform lesion in stage I of AOFVD is hyperautofluorescent on fundus autofluorescence (FAF) (**Figures 1**, **2**) (13). It is hypothesized that its hyperautofluorescence is derived from the autofluorescent precursors of lipofuscin (A2E, A2PE-H2, A2PE, and A2-rhodopsin), which supports histopathologic findings (46). In the pseudohypopyon stage, on FAF, the lesion will have a hyperautofluorescent inferior half with a hypoautofluorescent superior half (45). As the lesion progresses to the vitelliruptive and atrophic stages, the AVL disappears, and the lesion left behind is hypoautofluorescent due to underlying RPE atrophy (**Figure 2**) (45, 46).

### 3.3 Fluorescein angiography

On fluorescein angiography (FA), in the vitelliform stage, the lesion appears as a non-fluorescent central spot with a hyperfluorescent “spoked” ring without leakage (3, 10, 13, 47). Late phase images may demonstrate central staining of the lesion (**Figure 1**) (10). As the disease progresses towards the pseudohypopyon and vitelliruptive stages, the hyperfluorescence typically resolves, though a “stars-in-the-sky” appearance may be seen as focal nodules and cuticular drusen persist throughout these two stages (48). The stars-in-the-sky appearance and persistent hyperfluorescence in the vitelliruptive stage on FA and FAF may contribute to difficulty in distinguishing choroidal neovascularization (CNV). In these instances, other techniques, such as OCTA and indocyanine green angiography (ICGA), play an important role in establishing the diagnosis and planning treatment, especially for patients presenting at this stage for the first time in the clinic (14, 17).

### 3.4 Indocyanine green angiography

Battaglia et al. and Lanzetta et al. both characterized in 1996 the ICGA findings in eyes with AOFVD and vitelliform lesions (14, 53). ICGA reveals an oval, central dark spot beginning in the early frames of ICGA and persisting throughout all later frames (14, 53). The dark spot masks the underlying choroidal vessels. In later frames (between 8 to 15 minutes), a hyperindocyanescent area appears in approximately 81% of eyes (14, 53). The hyperindocyanescent area is irregularly round and located in the central area of the hypoindocyanescent dark spot. It is hypothesized that the blockage seen in the early frames is due to lipofuscin and melanin accumulation within the lesion, whereas the late frame hyperindocyanescence is evidence of more extensive RPE damage (53).

### 3.5 Spectral domain optical coherence tomography

**Figure 4:**
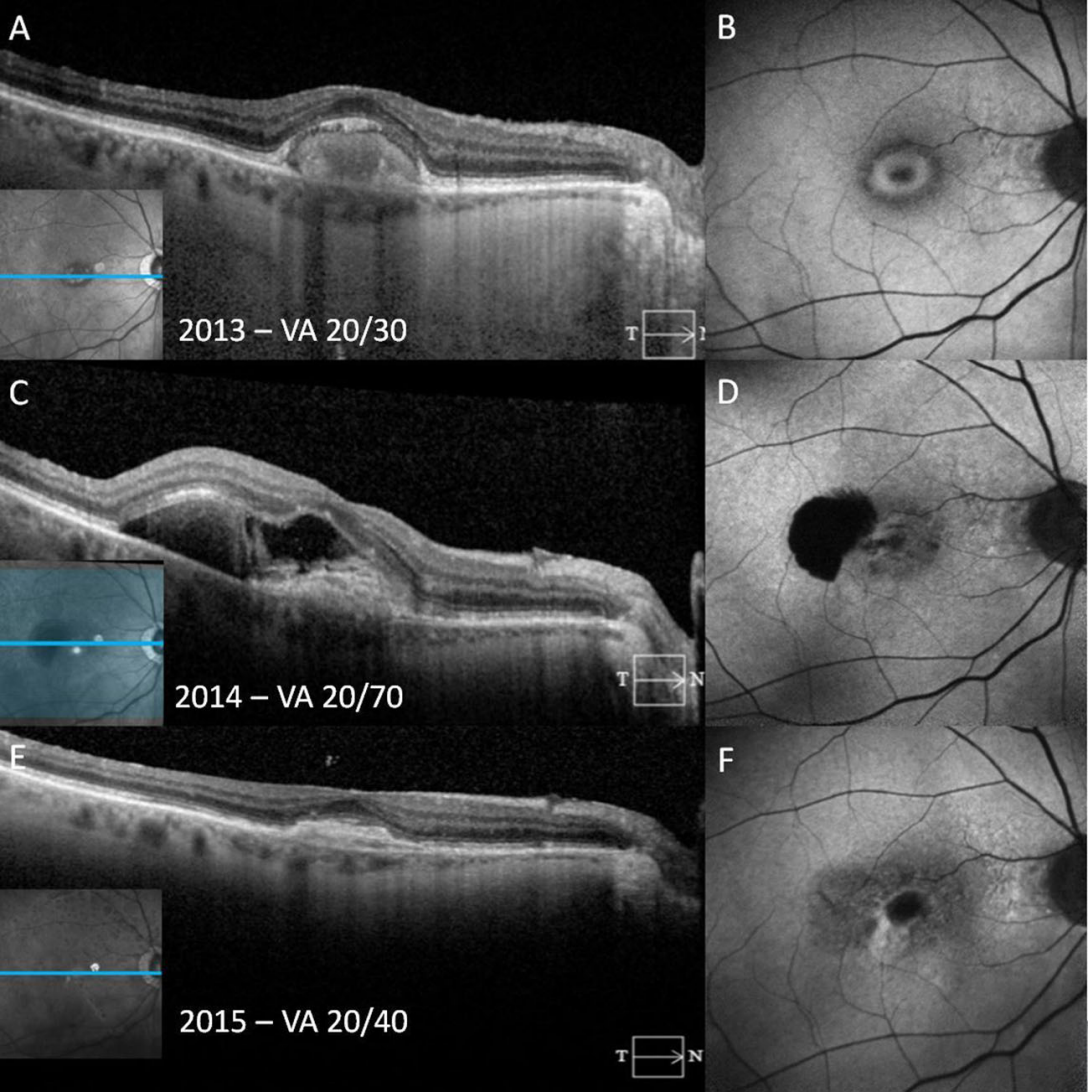
A patient with adult-onset foveomacular vitelliform dystrophy progresses to choroidal neovascularization over the course of a year. In the vitelliform stage, fundus autofluorescence (FAF) reveals the vitelliform lesion as a ring-shaped area of hyperautofluorescence with corresponding homogeneous subretinal hyperreflective material (SHRM) on spectral-domain optical coherence tomography (SD-OCT) **(A, B)**. One year later, SD-OCT shows an irregular SHRM with retinal pigment epithelium (RPE) elevation and relatively hyperreflective subretinal fluid (SRF) with a sharply demarcated juxtafoveal hypoautofluorescence secondary to both SHRM and an retinal pigment epithelium tear **(C, D)**. Following anti-vascular endothelial growth factor injections, photomicrographs show shallow hyperreflective RPE elevation and subretinal material consistent with a subretinal scar observed on SD-OCT and FAF **(E, F)**.

On SD-OCT, the vitelliform lesion corresponds to dome-shaped subretinal hyperreflective material (SHRM) between the RPE and ellipsoid zones in eyes with AOFVD in the vitelliform stage (**Figures 1**, **2** and **4**) (10, 16, 43). The ellipsoid zone is regularly disrupted in AOFVD, though this disruption may not impact VA (45). The height of the vitelliform lesion in AOFVD tends to grow more so than other AVLs, such as those due to AMD (45).

In all stages of AOFVD, there are frequently cuticular drusen at the RPE-Bruch’s membrane complex with associated RPE elevation, which may be intra- or extra-lesional (**Supplementary Figure 1**) (16, 46, 54). Querques et al. hypothesized that these cuticular drusen might represent RPE hyperplasia or macrophages containing large amounts of melanolipofuscin (16). Additionally, Wilde et al. describe that many patients (40%) newly presenting with AOFVD have subretinal drusenoid deposits (reticular pseudodrusen), which form secondary to damage in Bruch’s membrane and the RPE (48).

In the pseudohypopyon stage, the vitelliform lesion will consist of two different “zones.” In the upper zone, the material is relatively hyporeflective, with a few limited clumps of SHRM. The lower zone is characterized by SHRM. As in the vitelliform stage, the ellipsoid zone overlying the lesion is often disrupted (43). All other retinal layers from the internal limiting membrane to the external limiting membrane remain normal appearing (43). Intraretinal pseudocysts may also be seen during this stage. In the vitelliruptive stage, the lesion may appear on SD-OCT as if it is fragmented, with a mix of hyperreflective and hyporeflective spaces overlying the RPE (**Figure 2**). This process is frequently accompanied by overlying photoreceptor loss (16). As the vitelliruptive stage progresses, the lesion becomes progressively optically empty and hyporeflective with the hyperreflective material “clumping” and resolving along the RPE/photoreceptor interface (43). As the vitelliform lesion resolves, the overlying sensory retina may progressively attenuate, ultimately resulting in macular atrophy (**Figure 3**). CNV itself may present at any stage of AOFVD and will present with findings of subretinal fluid on SD-OCT (**Figure 4**).

### 3.6 Enhanced depth imaging

Only a few studies have utilized enhanced depth imaging (EDI) in the analysis of AOFVD, but these studies have helped elucidate some of the findings associated with each stage of disease. EDI demonstrates that the pseudohypopyon stage resembles more of a “croissant” configuration, with the lower hyporeflective space consisting of fluid (55). Additionally, studies have found that eyes in the pseudohypopyon stage have significantly increased choroidal thickness in the temporal and subfoveal regions (55, 56). Once eyes reached the vitelliruptive stage, increased choroidal thickness was seen by Grenga et al. across all locations in the retina (subfoveal, nasal, temporal, superior, and inferior regions) (56). This is in contrast to findings which support that choroidal thickness during the vitelliform stage is not significantly different from controls without AOFVD (55, 56).

### 3.7 Optical coherence tomography angiography

Optical coherence tomography angiography (OCTA) has emerged in the last 10 years as a new, non-invasive modality for assessing retinal blood vessel density and flow. Since 2016, many investigators have published reports which have characterized OCTA findings in patients with AOFVD.

Multiple studies have demonstrated a reduction in choriocapillaris vessel density in the foveal region corresponding to the AVL (19, 49, 51, 52). Cennamo et al. further elucidated that this reduction in choriocapillaris vessel density was seen only in the vitelliform and pseudohypopyon stages, whereas there was actually a significant increase in choriocapillaris vessel density during the vitelliruptive stage (19).

Multiple studies have demonstrated reduced blood flow in the superficial capillary plexus and deep capillary plexus in the foveal region corresponding to the vitelliform lesion (19, 49, 50). It is hypothesized that this is likely due to the shadowing effect from the vitelliform material, though it may be attributable to the mechanical compression of blood vessels by the vitelliform material, thus decreasing flow. Additionally, reduced flow in the choriocapillaris can be observed (19, 49, 50).

OCTA routinely demonstrates that patients with AOFVD possess an increased subfoveal choroidal thickness, which corroborates what has been reported on EDI (19, 49, 50). The choroidal thickness is increased compared to both healthy eyes as well as eyes with AMD (50). Both the increased choroidal thickness as well as reduced flow in the choriocapillaris supports that AOFVD possesses a pachychoroid, a feature which is defined by the attenuation of the choriocapillaris and dilation of choroidal veins (57). Further studies are needed in order to elucidate whether the decreased capillary plexus blood flow is an artifact of imaging.

Finally, OCTA may possess advantages over FA for the diagnosis of CNV in the context of AOFVD. As noted above, the vitelliform lesion may mask or be mistaken as neovascularization due to its late-phase staining in FA, with Joshi et al. identifying CNV in 1 eye out of 8 using OCTA, which had not been seen in FA (58). Additionally, Joshi et al. described a reduced hyporeflectivity in the vitelliform lesion area during the pseudohypopyon stage that is best appreciated by OCTA as opposed to FA (58). OCTA may thus be considered as an alternative to FA given its non-invasive approach and potentially more sensitive results.

## 4 Prognosis and complications

As described above, AOFVD frequently follows a relatively benign course, with most patients experiencing only a slight decline in BCVA (5, 13, 16, 59). Renner et al. reported that out of a cohort of 28 eyes followed between 1 to 5 years, 10 had no change in vision, 11 had a reduction and 4 patients had improved vision (13). Others have found that BCVA, in the absence of disease progression to CNV and macular atrophy, decreased only from 20/33 to 20/41 over an average of 7 years (17). There may also be color vision and visual field (VF) defects in as many as 50% of patients at baseline presentation, even in the absence of CNV or macular atrophy. Color vision defects are frequently severe (13). Other visual outcomes in patients with AOFVD may include an absolute scotoma and unstable or eccentric fixation, particularly with advanced stages of disease (59).

Importantly, there are sequelae of AOFVD that can significantly reduce VA., These include most notably CNV and macular atrophy (5, 16). Querques et al. demonstrated that stabilization of at the vitelliform stage corresponded to only a small change in BCVA (from 20/36 to 20/39), while progression to either the vitelliruptive or atrophic stages decreased BCVA from 20/50 to 20/104 (16). Other complications of AOFVD that have been reported include pigment epithelial detachments (PED), retinal folds, macular coloboma, and RPE aperture (60–63). Here, we expand on the prevalence of CNV and macular atrophy among patients with AOFVD, visual outcomes for those with CNV and macular atrophy, and risk factors for disease progression.

### 4.1 Choroidal neovascularization

The incidence of CNV in patients with AOFVD remains inadequately characterized with marked variance between the few estimates that are published, which may be secondary to differences in length of follow up between studies. Total incidence of CNV has been reported as being 2.1% (17), 7.7% (64), and 11.7% (65). Among papers which report rates of CNV, two describe the type of CNV. In both of these studies, all patients with CNV are described as having type 1 CNV (17, 65). For those patients with CNV, visual acuity is significantly affected. In Da Pozzo et al’s study of 51 eyes with AOFVD, VA was reduced from 20/80 to 20/250 among the 6 patients with CNV. Among the 3 eyes with diagnosed and treated CNV in Wilde et al’s study, there was an average change in BCVA -0.21 logMAR. Risk factors for CNV similarly remain poorly reported. Wilde et al. found a significant increase in risk for CNV with the presence of subretinal drusenoid deposits seen on OCT (64). Balaratnasingam et al. found that eyes with AVLs secondary to AMD had a greater risk of conversion to CNV than eyes with AVLs secondary to AOFVD (17). Other risk factors such as age, sex, and medical comorbidities are scarcely described, underscoring the need for research on this topic.

### 4.2 Macular atrophy

The incidence of macular atrophy in patients with AOFVD is perhaps less understood than in CNV. Balaratnasingam et al. reported an incidence of 21.3% in their cohort of 61 patients (17). Wilde et al. characterized the incidence of macular atrophy among a cohort of 26 patients with AVLs and found that 26.9% of individuals developed incident macular atrophy during the course of follow-up. Among those patients, 42% developed bilateral macular atrophy (64). Out of a larger prospective cohort of 237 eyes with AVLs, Chandra et al. found 21.9% exhibited macular atrophy at five years of follow-up (66).

Macular atrophy, unsurprisingly, has been associated with a significant decrease in vision, with studies reporting an associated decrease of up to 0.29 and 0.323 logMAR (17, 64). Increased risk of developing macular atrophy has been associated with patients who have lower baseline BCVA, greater maximum width of vitelliform lesions, and larger maximum height. Additionally, eyes that progress to the pseudohypopyon stage or vitelliruptive stage are more likely to progress towards macular atrophy (66). Similar to CNV, eyes with subretinal drusenoid deposits are also more likely to progress toward macular atrophy (64). Finally, similar to CNV, no studies have correlated patient or medical risk factors with conversion to macular atrophy.

### 4.3 Patient risk factors for progression

Apart from the risk factors described above for progression to macular atrophy or CNV, very few studies have identified risk factors associated with progression to the later stages of AOFVD. It remains unclear whether medical factors such as hypertension or chronic disease play any role in AOFVD or its progression. The only patient factor which is suggested to increase risk of progression is initial BCVA; worse baseline BCVA portends increased risk for macular atrophy and CNV (66).

## 5 Differential diagnoses

AOFVD is frequently misdiagnosed both due to unclear diagnostic standards and due to its close resemblance to other diseases. Here, we seek to provide clarity regarding the differentiation of AOFVD from two other vitelliform diseases: Best disease and nonneovascular AMD with vitelliform lesion.

### 5.1 Best disease

Adult onset vitelliform dystrophy shares many phenotypic features with Best disease. Both present with a vitelliform lesion that share the same features across fundus photography, FA, and SD-OCT (67). Best disease is also known as vitelliform macular dystrophy, which may lead to confusion when AOFVD is referred to as adult vitelliform macular dystrophy or adult vitelliform macular degeneration. The shared characteristics prompted Gass to propose in his original 1974 paper that AOFVD exists as a subtype of Best disease (1). Others have suggested that AOFVD is perhaps a more mild phenotype of Best disease (3). However, there are several distinct characteristics that are useful in distinguishing these two diseases.

AOFVD presents exclusively in adults, with the average age at diagnosis being in the 6^th^ decade of life (1, 3, 5, 13, 16). As described above, most patients diagnosed with AOFVD have a relatively benign disease course with preservation of VA. In contrast, Best disease is characterized by juvenile onset without loss of visual acuity until the sixth or seventh decades of life (25). Because many patients are not seen until they become symptomatic, it can be difficult to differentiate AOFVD from Best disease. In general, vitelliform lesions associated with Best disease are larger than those seen with AOFVD (68). That said, AOFVD may become quite large, so measuring lesion size is not a reliable outcome for differentiating the two diseases.

Best disease follows an AD inheritance pattern: most patients with Best disease will exhibit a missense mutation in *BEST1* (67). As discussed above, *BEST1* mutations have been associated with AOFVD, but they are not present in the majority of patients with AOFVD (31). Additionally, AOFVD has been shown to have a sporadic presentation. While there may be increased risk within families, it rarely presents in an AD fashion.

Prior studies have demonstrated that the most reliable way to differentiate between Best disease and AOFVD is by performing an electrooculogram (EOG). In Best disease, the EOG is abnormal with a reduced Arden’s ratio, whereas it is normal in the majority of patients with AOFVD (68).

### 5.2 Age-related macular degeneration with vitelliform lesions

AMD with vitelliform lesions is frequently difficult to differentiate from AOFVD. Patients present in the 6^th^ and 7^th^ decades of life for both diseases. Furthermore, because AOFVD patients are older, they frequently have comorbid drusen and subretinal drusenoid deposits (16, 48). Some studies define AMD with vitelliform lesions purely by the presence of an AVL with foveal drusen (46). However, this may be masking cases that might actually be AOFVD, which underscores the importance of strict diagnostic guidelines both for AMD with vitelliform lesions and for AOFVD.

Other studies differentiate AOFVD from AMD by the size of drusen (17). The consensus definition for AMD is drusen greater than 63 um with pigmentary abnormalities or large drusen (>125 um). The drusen must be present within 2 disc diameters of the fovea in persons older than 55 (69). Eyes with vitelliform lesions and drusen who do not meet these diagnostic standards should instead be diagnosed with AOFVD.

A recent study suggests that patients with AOFVD have greater macular choroidal thickness and subfoveal choroidal thickness than those with AMD, which may prove useful for clinical differentiation of the two diseases (70). Finally, AOFVD in the atrophic stage may be differentiated from AMD via the pattern of atrophy. In AOFVD, the atrophy will generally correspond to the area of the prior vitelliform lesion in the foveal region, whereas in AMD, atrophy may be diffuse and extrafoveal.

### 5.3 Pachyvitelliform maculopathy

Pachychoroid disease, as briefly discussed above, is a subset of retinal disease characterized by the dilatation of choroidal vessels in Haller’s layer and, to a lesser degree, in Sattler’s layer of the choroid. These vessel changes are often accompanied by choroidal filling defects and decreased flow within the choriocapillaris layer (57). Pachyvitelliform maculopathy is a subset of Pachychoroid disease where an acquired vitelliform lesion is observed in an area of enlarged pachyvessels. RPE dysfunction secondary to reduced flow within the choriocapillaris likely drives the development of the AVL (71, 72).

In pachyvitelliform maculopathy, the AVL may regress or migrate. Additionally, the AVL may resolve, only to reform in the same or different location (73). These phenomena are not typical of AOFVD. Additionally helpful in differentiating the two disease is the development of choroidal vessel dilation relative to the AVL. In, AOFVD, there is a reduction in choriocapillaris vessels in the vitelliform and pseudohypopyon stages of the disease, with a dilation of choroid vessels occurring in the vitelleruptive stage, suggesting that the AVL causes the pachyvessels (19). Finally, the association of AOFVD with mutations in genes known to be associated with the function of RPE cells further supports that the AVLs in AOFVD are not exclusively due to choroidal disease but rather primary RPE dysfunction (28, 33).

## 6 Treatment options

There are currently no treatments available to slow the progression of AOFVD. However, there are treatment options available for patients with sequelae of AOFVD such as CNV and macular atrophy (38).

For patients with CNV secondary to AOFVD, treatment with anti-vascular endothelial growth factors (anti-VEGF) therapies have proven useful and are standard of care. Studies published in support of this used bevacizumab (Avastin, Genentech) (74, 75) and ranibizumab (Lucentis, Genentech) (76–78). AOFVD patients with CNV who are treated with anti-VEGF medications may be able to preserve their vision longer than without treatment, with the majority of eyes showing stabilization of VA (74–76). Mimoun et al. found that 87.5% of patients treated with anti-VEGF injections lost fewer than 3 lines of vision one year following initiation of treatment (78).

Battaglia et al. explored the role of photodynamic therapy (PDT) in patients with pattern dystrophies and subfoveal CNV. Despite patients with other pattern dystrophies retaining baseline BCVA following PDT, patients with AOFVD demonstrated worsening of BCVA and no apparent response to PDT (79). Ergun et al. demonstrated similar results in patients with vitelliform lesions (80). As such, PDT does not currently have a role in treating patients with CNV and AOFVD.

For patients with macular atrophy, there are limited treatment options. There has been one case report which describes the results of a macular translocation for a patient with macular atrophy and AOFVD. Eckardt et al. attempted macular translocation with removal of vitelliform material in a 78-year-old woman with bilateral loss of vision secondary to AOFVD. Unfortunately, the patient experienced multiple retinal detachments following surgery as well as proliferative vitreoretinopathy. While her near vision improved slightly, there was no improvement from baseline at distance (81). The consensus following this procedure is that the risks of operation (and reoperation) did not outweigh the benefits (82). However, for patients with a macular hole, treatment with heavy silicone oil may effectively result in hole closure (83). New intraocular lens options such as the iolAMD EyeMax Mono are designed specifically for eyes with macular degeneration and aid in focusing images across a wider area directly onto the macula via a hyperspherical design (84). These lenses may maximize vision in patients with macular atrophy and AOFVD and may improve patients’ quality of life (85).

Finally, given that there might be a genetic component to AOFVD, patients may wonder if their family members should undergo genetic testing. AOFVD is a clinical diagnosis, and there are no formal guidelines for genetic testing in patients with AOFVD or their family members. Nevertheless, if multiple family members experience vision changes, it may warrant exploration with genetic testing. However, this is a topic that requires further research.

## 7 Discussion

AOFVD is a rare retinal disease with relatively limited literature regarding its expected progression and complications. Recent advances in imaging, particularly OCTA, may allow for a more sensitive assessment of complications like CNV in patients with AOFVD. Further studies which analyze risk factors for the development and progression of AOFVD would be of value in understanding this disease. By understanding risk factors for the development and progression of AOFVD, we may be more able to differentiate AOFVD from other disease entities that resemble it, such as Best disease and non-neovascular AMD.

## Author contributions

GN: Writing – original draft preparation, writing, review, and editing. TL: writing – review and editing. KS: writing – review and editing; image preparation. GM: writing – review and editing. MH: conceptualization; writing – review and editing; supervision. All authors contributed to the article and approved the submitted version.

## Supplementary material

Supplementary Figure 1
**(A, B)** Vertical spectral-domain optical coherence tomography (SD-OCT) shows pseudodrusens temporal to the fovea (white arrows) accompanied by subfoveal homogenous subretinal hyperreflective material (SRHM) overlying photoreceptor layer disruption (white star) with thick nodular retinal pigment epithelium (blue arrow).
