# Supplementary figures and images for: Adult-onset foveomacular vitelliform dystrophy: epidemiology, pathophysiology, imaging, and prognosis
Source: Front Ophthalmol (Lausanne). 2023 Aug 10;3:1237788. doi: 10.3389/fopht.2023.1237788 (PMC11182240; doi:10.3389/fopht.2023.1237788)

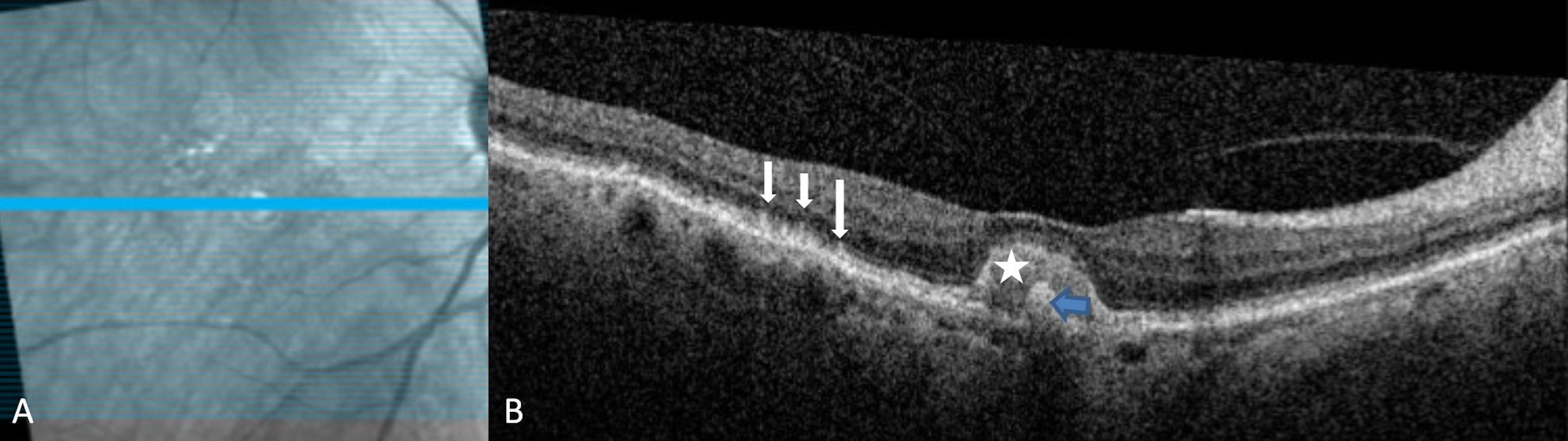

Supplement: Supplementary Figure 1 — (A, B) Vertical spectral-domain optical coherence tomography (SD-OCT) shows pseudodrusens temporal to the fovea (white arrows) accompanied by subfoveal homogenous subretinal hyperreflective material (SRHM) overlying photoreceptor layer disruption (white star) with thick nodular retinal pigment epithelium (blue arrow). [file Image_1.tiff]
